# Synthesis of nickel-boron/reduced graphene oxide for efficient and stable lithium-ion storage

**DOI:** 10.1016/j.heliyon.2024.e41074

**Published:** 2024-12-07

**Authors:** Gahyeon Im, Dami Yun, Hyun Bin Kim, Youn-Mook Lim, Seung-Hwan Oh, Huisu Kim, Byungnam Kim, KwangSup Eom, Jin-Mun Yun

**Affiliations:** a Radiation Fusion Research Division, Advanced Radiation Technology Institute (ARTI), Korea Atomic Energy Research Institute (KAERI), 29 Geumgu-gil, Jeongeup-si, Jeollabuk-do, 56212, Republic of Korea; b Advanced Battery Development Team 3, Hyundai Motor Company, Hwaseong, 18280, Republic of Korea; c School of Material Science & Engineering, Gwangju Institute of Science and Technology (GIST), 123 Cheomdangwagi-ro, Buk-gu, Gwangju, 61005, Republic of Korea

**Keywords:** Lithium-ion battery, Anode material, Boron, High capacity, Cyclic stability

## Abstract

Electrode material capacities and cycle performances must improve for large-scale applications such as energy storage systems. Numerous investigations have developed cathode materials to improve lithium-ion batteries (LIBs) performance: however, few have examined new anode materials. In this study, we synthesized a Ni-B/reduced graphene oxide (RGO) composites via a simple chemical reaction method to enhance the stability of electrodes in LIBs. A well-dispersed B, as a component of Ni-B composite, shortened the diffusion distance of lithium ion and allowed for the reversible storage and release of lithium ions. The incorporation of RGO significantly enhanced the dispersion of the Ni-B particles, preventing aggregation and enhancing the electrochemical performance. The long-term cyclic capacity of Ni-B/RGO reached approximately 1200 mAh g^−1^ at 400 mA g^−1^. Moreover, well-dispersed B from the reduction of B_2_O_3_ enhanced reactions with Li ions, gradually increasing the capacity. After several cycles, Ni-B/RGO maintained its structure without volume changes and with a uniform dispersion of elements. Therefore, Ni-B/RGO exhibited high stability over long cycles, leading to high reversibility. The combination of these features renders Ni-B/RGO a promising lithium storage material for LIBs.

## Highlights

• Synthesis of Ni-B/RGO anode using a simple chemical reduction method.
• Enhanced stability and performance of electrodes in lithium-ion batteries.
• High reversibility and long-term cyclic capacity of ∼1200 mAh g^−1^ at 400 mA g^−1^
• Efficient and stable lithium-ion battery anode material.

## 1 Introduction

With the increasing demand for sustainable energy, rechargeable lithium-ion batteries (LIBs) have become primary sources for portable devices and electric vehicles because of their lightweight, high energy density, and long durability [[1], [2], [3], [4], [5], [6]]. Advances in large-scale applications such as energy storage systems require substantial improvements in the capacities and cycle performances of electrode materials. However, several studies have focused on the development of cathode materials to enhance their performance, and few have investigated new anode materials for LIBs. The conventional anode material for LIBs is graphite (372 mAh g^−1^), known for its good stability and low working potential. However, achieving higher capacities to meet the requirements of high-energy and high-power-density applications remains challenging [7,8]. Consequently, conversion-type anode materials with higher capacities than graphite-based anodes are attractive as promising alternatives to overcome these limitations [[9], [10], [11]].

Among these, B is lightweight and achieves a superior theoretical capacity (2479 to 12,395 mAh g^−1^) owing to its potential to theoretically form approximately five bonds with a Li ion [12]. Despite this significant advantage, B-based materials have not been widely used because of their low electrochemical reactivity, which hinders their direct reactions with Li ions. Additionally, B-based materials have strong covalent bonds, limiting their participation in electrochemical reactions [[13], [14], [15]]. Therefore, B activation is crucial for the use of B-based materials as anodes in LIBs.

Various approaches have been proposed to address these objectives. *Liu* et al. fabricated a Li-B alloy and used it as an anode in LIBs. However, the achieved discharge capacity (660 mAh g^−1^) was not recovered owing to unstable B [16]. *Dong* et al. modified B oxide by designing a B_2_O_3_/Fe composite for B materials [12]. Despite superior capacities being achieved at a high rate, this method reduces active materials because high heat treatments below 500 °C are required to obtain final compounds. Numerous studies have employed a pulse laser deposition (PLD) method to fabricate a tetragonal B (B_50_) thin-film electrode [17]; however, this method is challenging to commercialize. Therefore, a simple and efficient approach for fabricating uniform B-based compounds with enhanced performances and capacities is crucial.

Considering the conductivity effects of materials on electrochemical performance, a transition metal can accelerate Li ion accommodation in oxide species [[18], [19], [20], [21]]. First, we propose a transition metal to activate B-based materials by accelerating the accommodation of Li ions and weakening strong non-metallic covalent bonds. Owing to its low cost and reversible redox reactions, we selected Ni, which is extensively studied in the fields of magnetics and supercapacitors [[22], [23], [24], [25]]. Then, Ni-B composites were prepared, where Ni-activated B to accommodate Li ions [13,26,27]. However, the Ni-B particles aggregated and caused volume expansion during cycling, impeding their Li-ion storage capabilities and electrochemical performance [14]. Therefore, we used reduced graphene oxide (RGO) as a support to disperse particle aggregation and alleviate volume expansion while maintaining electrode stability. Moreover, the metallic behavior of RGO enhances the electrochemical performance of the active materials [[28], [29], [30], [31], [32]].

In this study, we designed Ni-B/RGO composites as anode materials by combining the advantages of Ni and RGO. A well-dispersed B shortened the diffusion distance of Li ions and allowed for the reversible storage and release of Li ions. The RGO ensures stability by providing a uniform dispersion of Ni-B and alleviating volume changes. Based on these properties, we demonstrated that the Ni-B/RGO composite exhibited a high reversible capacity and outstanding cyclic stability in LIBs.

## 2 Experimental

### 2.1 Material synthesis

A simple chemical reduction method was employed to synthesize the Ni-B/RGO material. The reagents were purchased from Sigma Aldrich and used without further purification. First, NiCl_2_·6H_2_O precursor (0.5 g) was dissolved in 10 mL of deionized (DI) water under constant magnetic stirring. Next, the prepared solution was mixed with 100 mL of graphene oxide dispersion (5 mg/mL in DI water) at room temperature. After brief sonication, NaBH_4_ aqueous solution (40 mL) was slowly added to the mixture to proceed with the reduction reaction. Consequently, the color of the solution changed to black when the NaBH_4_ solution was added dropwise, simultaneously generating H_2_ gas. The precipitate was collected by centrifugation and washed with DI water. The final product was freeze-dried for 48 h. The method used to prepare the Ni-B particles was similar, with the exception that graphene oxide was not added.

For the preparation of the B/RGO composite, B powder (Merck, Cas NO. 7440-42-8) was mixed with RGO prepared by the NaBH_4_ reducing agent with a weight ratio of 1:1.

### 2.2 Materials characterization

A scanning electron microscope (SEM; *S-4700* EMAX system, *Hitachi*) equipped with an energy-dispersive spectrometer (EDS) was employed to observe the morphological properties of the materials. Scanning transmission electron microscopy (STEM; *Tecnai G2 F30 S-Twin, FEI*), field-emission transmission electron microscopy (FE-TEM; *JEM-2100F, JEOL*), and selected-area electron diffraction (SEAD) were also conducted. Powder X-ray diffraction (XRD; Alpha-1, *PANalytical*) patterns were measured at a scanning rate of 2° min^−1^ in the 2θ range of 10–80°. X-ray photoelectron spectroscopy (XPS; K-Alpha, *Thermo Fisher*) with Al Kα radiation was employed to examine the chemical composition. Thermo Avantage software was used to conduct the convolution and characterization of the XPS spectra.

### 2.3 Fabrication of electrodes

Ni-B, B/RGO, and Ni-B/RGO, activated carbon (*SuperP, TIMCAL*), and polyvinylidene fluoride (PVDF, *MTI*) were mixed in *N*-methyl pyrrolidone (NMP, *Sigma Aldrich*) solution at a weight ratio of 80:10:10 to produce a slurry. The slurry was uniformly coated onto a Cu foil. Next, the fabricated electrodes were dried overnight at 80 °C in a vacuum oven while the electrodes served as working electrodes. The loading mass of active material in the anode electrode for cycling is 0.53 mg cm^−2^.

### 2.4 Electrochemical measurements

Half cells (CR2032 coin type, *MTI*) were assembled with the as-prepared working electrode, Li chips (16 Φ) as a counter electrode, Celgard 2400 separator, and 1.0 M LiPF_6_ in a 1:1 v/v mixture of ethylene carbonate:diethyl carbonate as an electrolyte in an Ar-filled glove box. The glove box maintained moisture and O contents below 1 ppm. A cycler test system (*WBCS 3000S, WonATech*) was employed to conduct electrochemical measurements at room temperature. The discharge–charge tests of the cells were performed within a voltage window of 0.05–3.00 V_Li_^+^_/Li_. The rate capabilities were evaluated at various current densities. A cycler (*WBCS 3000S, WonATech*) was employed to conduct cyclic voltammetry (CV) tests at a scanning rate of 0.1 mV s^−1^ over the potential range of 0.01–3.00 V_Li_^+^_/Li_. An impedance analyzer (*Solartron analytical, 1260*) connected to a potentiostat (*Solartron analytical, 1287*) 0.01 Hz at 1.0 MHz frequency ranging from 0.005 V at various potentials was employed to perform electrochemical impedance spectroscopy (EIS) analyses. An impedance-fitting program (*Z-view, Scribner Associate*) was employed to analyze the Nyquist values.

## 3 Results and discussion

### 3.1 Materials characterizations

**Fig. 1:**
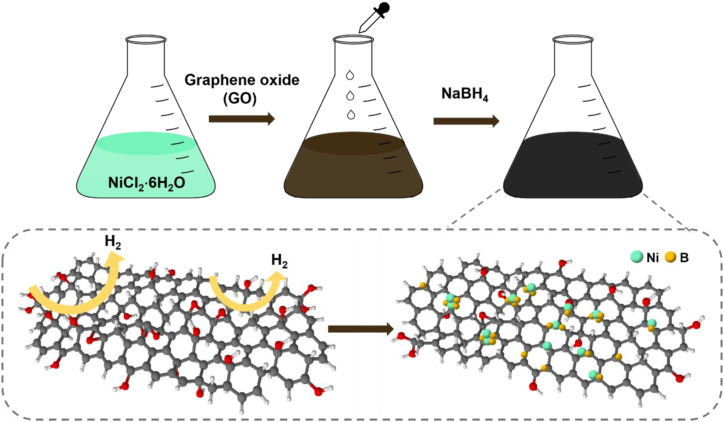
Synthetic route of the Ni-B/RGO anode material via a chemical reduction method.

Fig. 1 illustrates the preparation of Ni-B/RGO via a simple chemical reduction method. Initially, a GO/NiCl_2_·6H_2_O solution was prepared by physical mixing to maintain uniformity. Next, NaBH_4_ aqueous solutions, serving as sources of B and reductants for GO, were slowly added, generating H_2_ gas and turning the solution black owing to GO reduction. This method offers a straightforward synthesis by forming Ni-B particles and converting GO to RGO simultaneously.

**Fig. 2:**
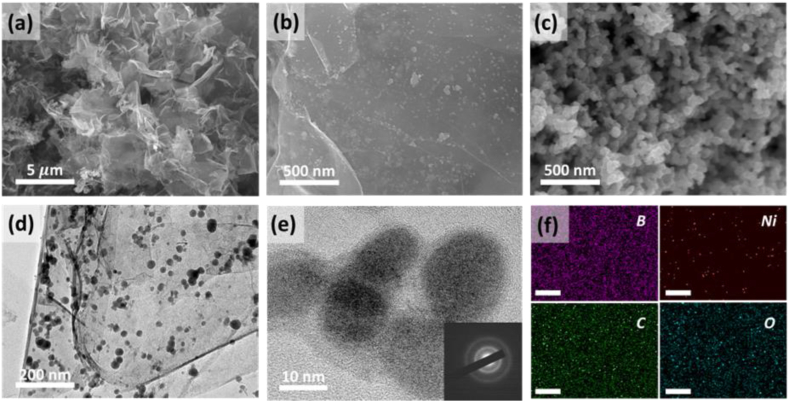
Morphologies of Ni-B/RGO composite: (a–b) Scanning electron microscope (SEM) surface image of Ni-B/RGO and (c) Ni-B particles. (d–e) Field emission transmission electron microscopy (FE-TEM) of Ni-B/RGO. (e) Ring pattern collected by selected area electron diffraction. (f) Elemental mappings of Ni-B/RGO for B, Ni, C, and O species.

**Fig. 3:**
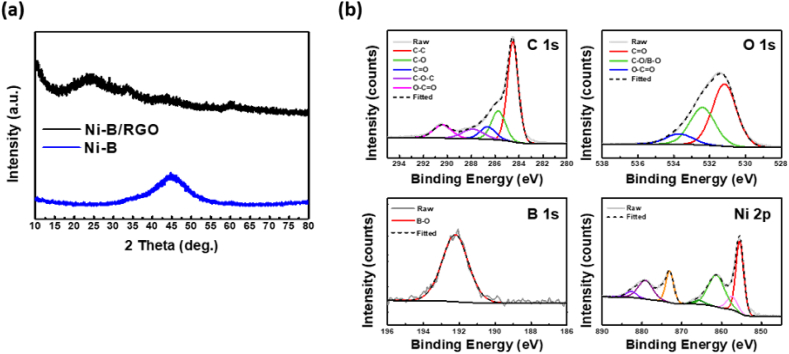
Chemical structure of Ni-B/RGO: (a) X-ray diffraction (XRD) pattern for Ni-B/RGO and Ni-B samples. (b) X-ray photoelectron spectroscopy (XPS) analysis for C 1s, O 1s, B 1s, and Ni 2p of the Ni-B/RGO sample.

Fig. 2 shows the morphological properties of Ni-B and Ni-B/RGO. The Ni and B were uniformly distributed on the RGO surface, as shown in Fig. 2a and b, with Ni-B particles exhibiting a nanosized morphology and broad size distribution from 20 to 40 nm. In contrast, the pure Ni-B nanoparticles synthesized without GO tended to aggregate, as shown in Fig. 2c. The RGO in the Ni-B/RGO composite effectively prevented Ni-B nanoparticle agglomeration, mitigating capacity decay during cycling tests. The TEM images show that uniform Ni-B nanoparticles were formed on the RGO surface, as shown in Fig. 2d. Additionally, elemental mapping further confirmed this uniform distribution, as shown in Fig. 2f. TEM (Fig. 2e) and XRD (Fig. 3a) were employed to confirm the amorphous structure of the materials. The overall XRD patterns showed no crystalline peaks, indicating the amorphous structure of Ni-B/RGO. The observed broad diffraction peak at approximately 25° was attributable to RGO [33], and the weak and broad pattern at approximately 45° was identical to the pattern of the Ni-B particles, confirming that Ni-B/RGO is a composite of RGO and Ni-B particles. Furthermore, XPS analysis revealed the spectrum of C 1s, displaying characteristic peaks at 284.0, 285.3, 286.5, 287.8, and 289.9 eV, corresponding to C-C, C-O, C-O-C, C=O, and O-C=O species, respectively, as shown in Fig. 3b. Decreased peak intensity associated with the O-functionalized groups of GO indicated the conversion of GO to RGO by removing the O groups of pristine GO, which was confirmed by the XRD results [34]. The O 1s peaks at 530.6, 531.9, and 533.19 eV corresponded to the C=O, C-O, and O-C=O species, respectively. Two main peaks of Ni 2p_3/2_ and Ni 2p_1/2_, located at 855.4 and 873.1 eV, confirmed the presence of Ni^2+^. Moreover, the B 1s spectrum exhibited a prominent peak at 191.73 eV, assigned to the BO bond [35,36]. Therefore, the synthesis of an amorphous Ni-B/RGO composite comprising RGO and Ni-B with B-O and Ni-O bonds was confirmed.

### 3.2 Electrochemical behavior of Ni-B/RGO anodes

**Fig. 4:**
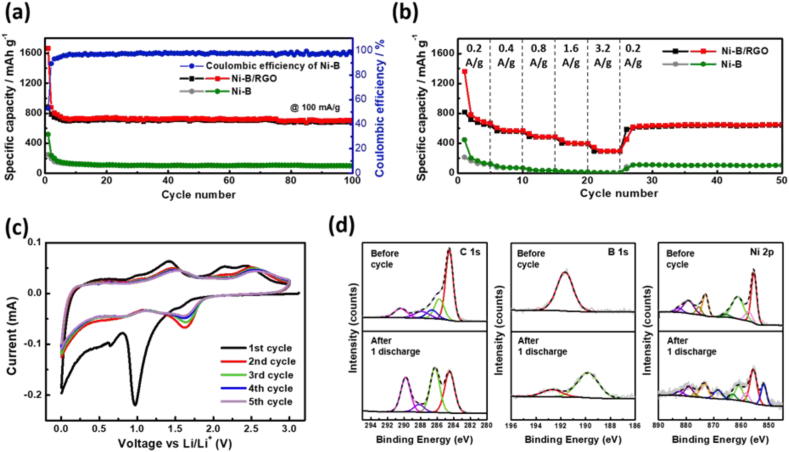
Electrochemical behaviors and post-mortem analysis of Ni-B/RGO: (a) Cycle performances of Ni-B/RGO and Ni-B (b) Rate performances at various rates from 200 to 3200 mAg^−1^; the charge–discharge tests were conducted in a range of 0.05–3.0 V. (c) Cyclic voltammetry (CV) for the initial 5 cycles of a Ni-B/RGO half-cell; CV were conducted with an operating voltage of 0.05–3.0 V_Li_^+^_/Li_ at 0.1 mV s^−1^. (d) X-ray photoelectron spectroscopy (XPS) analysis (C 1s, B 1s, and Ni 2p) of Ni-B/RGO electrode before and after the first discharge process.

The electrochemical performance of the synthesized Ni-B/RGO electrode was evaluated using a half-cell configuration and compared with the Ni-B electrode. The Ni-B/RGO electrode yielded an initial discharge capacity of 1665 mAh g^−1^, significantly surpassing that of the Ni-B-only electrode, yielding a capacity of 517 mAh g^−1^, as shown in Fig. 4a. The electrodes exhibited enhanced capacities compared to those of B/RGO material (Fig. S1), indicating that conductive Ni can activate inert B. The discharge–charge profiles of Ni-B/RGO displayed stable curves (Fig. S2a), maintaining a high reversible capacity without significant changes, which was consistent with the cyclic performance results.

In contrast, the Ni-B electrode experienced significant capacity decay during repeated cycling tests (Fig. S2b) owing to the aggregation of active materials. Moreover, it increased Li-ion diffusion paths, leading to continuous volume expansions, hindering Li-ion diffusion, and yielding poor performance [37,38]. Therefore, the enhanced cycling performance of Ni-B/RGO was attributable to its structural ability to alleviate agglomeration and volume changes.

To further investigate the rate capability of Ni-B/RGO and Ni-B, we changed the charging–discharging rates (200, 400, 800, 1600, and 3200 mA g^−1^) and fixed them at 200 mA g^−1^ for subsequent cycles to evaluate the rate capability of the Ni-B/RGO and Ni-B anodes for half-cells (Fig. 4b). Similarly, the Ni-B/RGO electrode exhibited superior capacity retention and performance across various current densities than pure Ni-B without RGO.

CV was conducted in the potential window 0.01–3.0 V at a scan rate of 0.1 mV s^−1^ to investigate the redox reaction mechanisms of Ni-B/RGO (Fig. 4c). In the initial step, Ni-B/RGO displayed two characteristic peaks: a strong reduction peak at 0.97 V, attributable to the insertion of Li into B_2_O_3_ (Eq. (1)) [12]. Although Eq. (1) dose not clearly demonstrate how the lithiation process could be reversible, recently published work has confirmed that during the lithiation/discharge process, there is a gradual increase in the percentage of Ni^2+^ and a decrease in the percentage of Ni^3+^ [39]. Thus, Li forms bonds with B atoms and transfer electrons to Ni atoms, converting Ni^3+^ into Ni^2+^. Second, a n additional peak at 0.47 V corresponded to the reduction of Ni^2+^ to Ni^0^ with the formation of Li_2_O (Eq. (2)) [40,41]. Transition metal Ni provides a conductive matrix that activates inert B materials and facilitates Li-ion accommodation in the B materials. In a subsequent wide sweep in 0–0.5 V range, a peak associated with the reaction of B with Li for forming Li_x_B (x = 1−5) was observed (Eq. (3)) [42]. Irreversible reactions in this range resulted from electrolyte decomposition with the formation of the solid electrolyte interphase(SEI) layer during the first cycle [40,43]. The two main reduction peaks at 0.97 and 0.47 V shifted to 1.60 and 0.82 V from the second cycle, respectively, owing to electrode activation, facilitating seamless reactions with Li. During delithiation, two oxidation peaks were observable at 1.42 and 2.51 V, corresponding to organic SEI layer dissolution and the reverse reaction of Eq. (2). An additional oxidation peak at 2.10 V was observable from the oxidation of B [44]. After the second sweep, the CV profiles overlapped, indicating good reversibility of the reactions of B_2_O_3_, NiO, and B (Eqs. (Equation 1), (Equation 2), (Equation 3))). Comparatively, the CV profiles of Ni-B and pure B were also tested and exhibited poor reversibility (Figs. S2 and S3).

(Equation 1) B_2_O_3_ + Li^+^ + xe^−^ ↔ Li_x_B_2_O_3_

(Equation 2) NiO + 2Li^+^ + 2e^−^ ↔ Ni + Li_2_O

(Equation 3) B + xLi^+^ + xe^−^ ↔ Li_X_B

In this study, we conducted an XPS analysis to confirm this reaction, demonstrating the changes in the binding energy after the discharging steps, as shown in Fig. 4d. The B 1s binding energy before cycling was approximately 192 eV, indicating the presence of BO bonds in B_2_O_3_. After the first discharge to 0.05 V, the intensity of the peak at 192 eV weakened, and a new signal was observable at 190 eV, suggesting that the valence state of B diminished from B_2_O_3_ to a lower state (Li_x_B_2_O_3_, B, Li_x_B). This result supported the reduction of B_2_O_3_. In addition, a Ni^0^ peak at 851 eV was observable owing to the reduction of Ni^2+^ to Ni^0^ according to the discharge reaction. Furthermore, the increased C 1s peaks at 285–286 and 290 eV, corresponding to carbonate compounds (C=O and C-O bonds), indicate SEI layer formation from electrolyte decomposition [45].

### 3.3 Long-term cyclic performance of Ni-B/RGO anodes

**Fig. 5:**
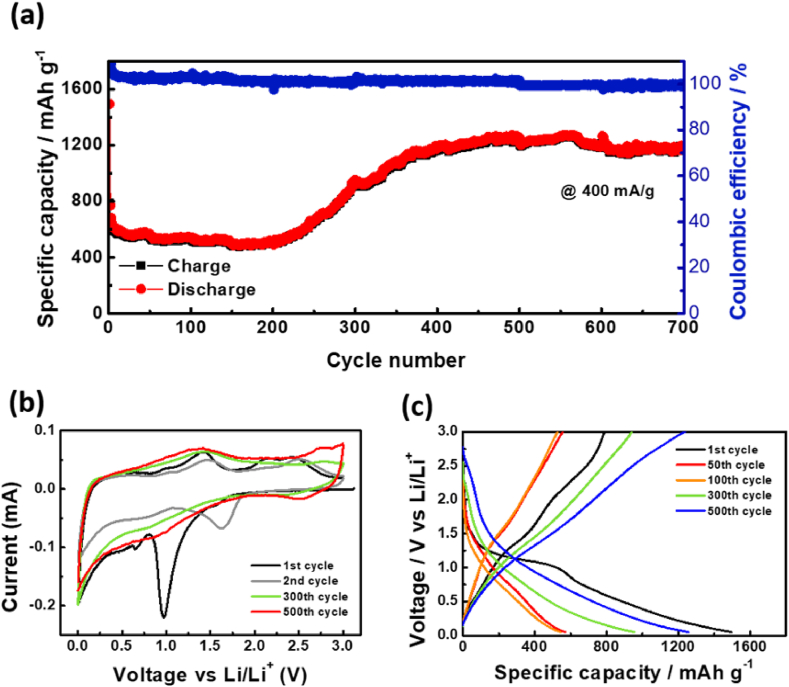
Representative long-term electrochemical behavior of Ni-B/RGO: (a) Cycle performances of Ni-B/RGO for 700 cycles. The charge–discharge test was conducted in a range of 0.05–3.0 V_Li_^+^_/Li_. (b) Cyclic voltammetry (CV) after the 1st, 2nd, 300th, and 500th charge–discharge cycles at a scanning rate of 0.1 mV s^−1^. (c) Charge–discharge profiles of Ni-B/RGO at the 1st, 50th, 100th, 300th and 500th charge–discharge cycles with an operating voltage of 0.05–3.0 V.

The long-term performance of Ni-B/RGO was measured at 400 mA g^−1^ for 700 cycles in a range of 3.0 to 0.05 V_Li+/Li_, as shown in Fig. 5a. The capacity gradually increased to 500 cycles at 400 mA g^−1^ and reaching approximately 1200 mAh g^−1^. To analyze the performance in which the capacity of Ni-B/RGO gradually increased during cycling, CV measurements with long-term cycles (at the 300th and 500th cycles) were performed, as shown in Fig. 5b. The peak at 1.60 V diminished, while the peak at 0.82, which indicates the redox reactions of B_2_O_3_ (Eq. (1)) at 1.60 V weakened. However, reactions at 0.62 V related to NiO (Eq. (2)) were enhanced. A previous study [12] suggested that O from B_2_O_3_ may be transferred to NiO when B_2_O_3_ is reduced to B during repetitive cycling. Therefore, the redox reactions of NiO at 0.62 V (discharge cycle), 1.42 V, and 2.51 V (charge cycle) were gradually enhanced. Furthermore, B can participate strongly in electrochemical reactions, as indicated by the intense peak near 0.5 V. This peak at approximately 0.5 V was connected to the main capacities, indicating that the increased capacities of long-term cycles originated from the plateau below 0.5 V, as shown in Fig. 5c.

**Fig. 6:**
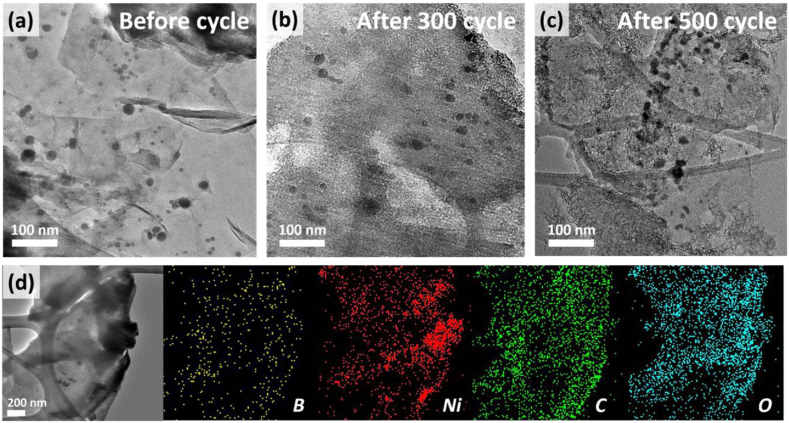
TEM images before and after long-term charge–discharge cycles (300 and 500) and elemental mapping of Ni-B/RGO after 500 cycles.

TEM was employed to examine the morphological changes in the active materials after long-term cycling, as shown in Fig. 6. Compared to alloying/conversion anode materials that underwent significant volume expansion, the Ni-B/RGO structure was maintained and supported by the RGO structure, mitigating the volume expansion. Fig. 6d shows the nanosized Ni and B particles that remained uniformly distributed on RGO after 500 cycles, as confirmed by EDS mapping. The uniform distributions of B and Ni particles on RGO ensured smooth reactions with Li ions, gradually increasing the capacity. CV analysis demonstrated that the dispersed B formed from the reduction of B_2_O_3_ significantly increased the capacity by promoting Li storage in B.

**Fig. 7:**
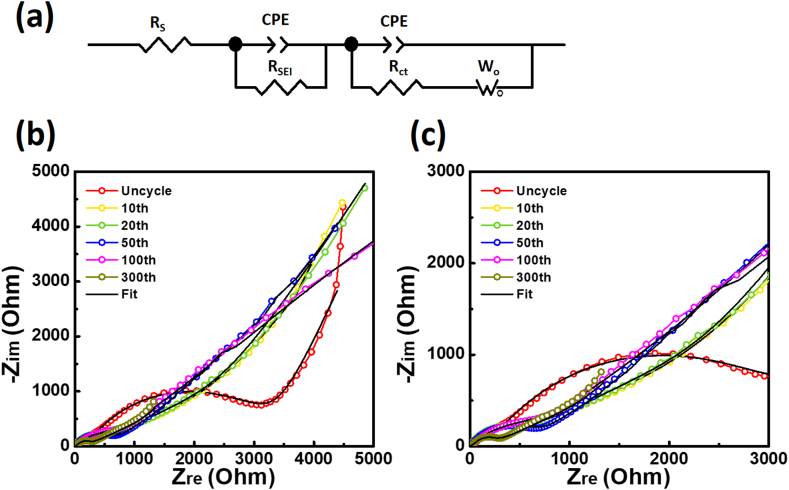
Electrochemical impedance spectroscopy (EIS) of Ni-B/RGO: (a) Equivalent circuits for Ni-B/RGO half cell. R_s_ is the electrolyte resistance. R_ct_ is the charge transfer resistance. R_SEI_ is the resistance of SEI layer. CPE are constant phase elements of the SEI and the electrode. W_o_ is the open Warburg element. (b) Nyquist plots of Ni-B/RGO electrode before any cycle and after 10, 20, 50, 100, 300 cycles.

Impedance analysis was conducted to evaluate the influence of the internal resistance of the electrodes (Fig. 7). Nyquist plots of the Ni-B/RGO electrode and equivalent circuits were recorded before and after 10, 20, 50, 100, and 300th cycles. The plots show semicircles at high frequencies and straight slope lines at low frequencies. The high-frequency semicircle corresponds to the resistance (R_s_, R_sei_) and capacitance (C_sei_) associated with the SEI layer formation. However, the medium-frequency semicircle corresponds to the charge transfer resistance (R_ct_) and double-layer capacitance (CPE). The low-frequency straight slope represents the Warburg element (W_o_) from Li-ion diffusion [46]. The semicircle diameter decreased rapidly after the first cycle, indicating a reduction in R_ct_ owing to the activation effect of the electrode in subsequent cycles (Fig. 7b). The R_ct_ value decreased from 3049–325.8 Ω, corresponding to the results of Nyquist plots, as summarized in Table 1. R_ct_ of the cell decreased in the tenth cycle. However, the resistance in subsequent cycles increased to 771.1 Ω.

**Table 1:** Electrochemical impedance spectroscopy (EIS) fitting results after the 10th, 20th 50th, 100th, and 300th cycles for Ni-B/RGO half-cell: Fitted results of each resistance (R_S_, R_SEI_, and R_ct_) are calculated from the proposed equivalent circuit for Ni-B/RGO (Fig. S4a), where R_s_ is the electrolyte resistance, R_film_ is the film resistance, R_ct_ is the charge transfer resistance, W_o_ is the Warburg resistance, CPE are constant phase elements of the SEI and the electrode.

	R_s_	R_sei_	R_ct_
Before cycle	8.14	230.80	3049
10th Cycle	6.49	32.23	325.8
20th Cycle	8.08	31.31	375.7
50th Cycle	4.92	22.40	401.5
100th Cycle	17.50	40.12	771.1
300th Cycle	4.12	63.76	127.4

Conversely, R_ct_ decreased to 127.4 Ω after long-term 300 cycles. Additionally, B was formed by the reduction of B_2_O_3,_ which was dispersed on conductive Ni and RGO over long cycles, yielding enhanced storage of Li ions in B. In addition, the Li-ion diffusivity in the active material increased gradually, decreasing R_ct_. The activation effect of B and the increase in diffusivity had synergistic effects on the electrochemical performance of Ni-B/RGO.

## 4 Conclusion

In this study, we successfully synthesized Ni-B/RGO composites via a simple chemical reaction method for efficient and stable Li-ion battery applications. The incorporation of RGO significantly enhanced the dispersion of the Ni-B particles, preventing aggregation and enhancing the electrochemical performance. Ni-B/RGO exhibited superior capacities than pure Ni-B, with a higher retention of approximately 100 % and good reversibility originating from the redox mechanisms of B_2_O_3_, NiO, and B. The long-term cyclic performance exhibited increased capacity, reaching approximately 1200 mAh g^−1^ at 400 mA g^−1^ without significant volume change. Thus, we demonstrated that Ni activates B-based materials, promoting Li storage. However, RGO maintained structural stability by mitigating particle agglomeration and volume expansion. Additionally, B formed from the reduction of B_2_O_3_ dispersed well on Ni and RGO over long-term cycling, enhancing the reactions with Li ions and increasing the diffusivity of Li^+^. Therefore, Ni-B/RGO exhibits high stability over long cycles with high reversibility. Consequently, the Ni-B/RGO anode material is a promising candidate for Li-ion battery applications. Further studies are required to elucidate the specific mechanisms of Ni-B/RGO owing to its potential use as an anode material in LIBs.

## CRediT authorship contribution statement

**Gahyeon Im:** Writing – original draft, Data curation, Conceptualization. **Dami Yun:** Writing – original draft, Data curation, Conceptualization. **Hyun Bin Kim:** Project administration, Funding acquisition. **Youn-Mook Lim:** Formal analysis, Data curation. **Seung-Hwan Oh:** Formal analysis, Data curation. **Huisu Kim:** Software, Resources. **Byungnam Kim:** Software, Resources. **KwangSup Eom:** Writing – review & editing, Supervision. **Jin-Mun Yun:** Writing – review & editing, Supervision.

## Data availability

The datasets used and/or analyzed during the current study available from the corresponding author on reasonable request.

## Funding

This study was supported by the 10.13039/501100003720Korea Atomic Energy Research Institute (KAERI) Institutional Program (Project No. 2710007330).

## Declaration of competing interest

The authors declare that they have no known competing financial interests or personal relationships that could have appeared to influence the work reported in this paper.
