# Supplementary material for: Synthesis of nickel-boron/reduced graphene oxide for efficient and stable lithium-ion storage
Source: Heliyon. 2024 Dec 7;10(24):e41074. doi: 10.1016/j.heliyon.2024.e41074 (PMC11696628; doi:10.1016/j.heliyon.2024.e41074)
Supplement: Multimedia component 1 [file mmc1.docx]

**Supplementary Materials**

**Synthesis of Nickel-Boron/Reduced Graphene Oxide for Efficient and Stable Lithium-Ion Storage**

Gahyeon Im^b^, Dami Yun^a,c^, Hyun Bin Kim^a^, Youn-Mook Lim^a^, Seung-Hwan Oh^a^, Huisu Kim^a^, Byungnam Kim^a^, KwangSup Eom^c,*^ and Jin-Mun Yun^a,^^[[1]](#footnote-1)^

*^a^Radiation Fusion Research Division, Advanced Radiation Technology Institute (ARTI), Korea Atomic Energy Research Institute (KAERI), 29 Geumgu-gil, Jeongeup-si, Jeollabuk-do 56212, Republic of Korea*

*^b^Advanced Battery Development Team 3, Hyundai Motor Company, Hwaseong, 18280 Republic of Korea*

*^c^School of Material Science & Engineering, Gwangju Institute of Science and Technology (GIST), 123 Cheomdangwagi-ro, Buk-gu, Gwangju 61005, Republic of Korea*

**Tables**

**Table S1. Elemental contents of the Ni-B/RGO composites.**

| **Element** | **Weight %** | **Atomic %** |
| --- | --- | --- |
| B | 6.52 | 15.36 |
| C | 11.24 | 23.83 |
| O | 21.71 | 34.55 |
| Ni | 60.53 | 26.25 |

**Table S2. Summary of the electrochemical performance of the boron-based anode materials**

| **Materials** | **Reversible capacity(mAh g^-1^)** | **Capacity retention**  **(cycle #, current density)** | **Ref.** |
| --- | --- | --- | --- |
| PB22 | 679.2 | - | [S1] |
| CB1 | 280.4 | - | [S1] |
| B-MPCFs | 340 | - | [S2] |
| B-HPSi | 1800 | 93% (150 cycles, 0.84 A g^-1^) | [S3] |
| B-Gr | 235 | 79.2% (30 cycles, 50 mA g^-1^) | [S4] |
| B_2_O_3_-SnO_2_/G | 2283.3 | No obvious loss  (200 cycles, 500 mA g^-1^) | [S5] |
| NBGs-1000 | 429 | 96.5% (125 cycles, 50 mA g^-1^) | [S6] |
| rGO-Co-B-2 | 894 | No obvious loss  (500 cycles, 1000 mA g^-1^) | [S7] |
| AFBO | 1250 | NO loss  (250 cycles, 0.5 & 1.0 A g^-1^) | [S8] |
| Boron nanorod | 170 | 83.47% (500cycles, 55 mA g^-1^) | [S9] |
| Ni-B/RGO | 650 | NO loss  (700 cycles, 400 mA g^-1^) | This work |

[S1] G. Yin, Y. Gao, P. Shi, X. Cheng, A. Aramata, *Mater. Chem. Phys*. **2003**, *80*, 94.

[S2] Y. Nishimura, T. Takahashi, T. Tamaki, M. Endo, M. S. Dresselhaus, *TANSO* **1996**, *172*, 89.

[S3] Y. Ren, X. Zhou, J. Tang, J. Ding, S. Chen, J. Zhang, T. Hu, X. Yang, X. Wang, J. Yang, *Inorg. Chem*. **2019**, *58*, 4592.

[S4] Z.-S. Wu, W. Ren, L. Xu, F. Li, H.-M. Cheng, *ACS Nano* **2011**, *5*, 5463.

[S5] L. Wen, X. Qin, W. Meng, N. Cao, Z. Song, *Mater. Sci. Eng*. *B* **2016**, *213*, 63.

[S6] S. Huang, L. Zhang, J. Zhu, S. P. Jiang, P. K. Shen, *J. Mater. Chem. A* **2016**, *4*, 14155.

[S7] D. Wang, J. Zhou, J. Li, X. Jiang, Y. Wang, F. Gao, *Chem. Eng. J*. **2019**, *360*, 271.

[S8] W. Dong, Y. Zhao, X. Wang, X. Yuan, K. Bu, C. Dong, R. Wang, F. Huang, *Adv. Mater*. **2018**, *30*, 1801409.

[S9] C. Deng, M. L. Lau, H. M. Barkholtz, H. Xu, R. Parrish, M. Xu, T. Xu, Y. Liu, H. Wang, J. G. Connell, K. A. Smith, H. Xiong, *Nanoscale* **2017**, *9*, 10757.

**Figures**

**Fig. S1. Cyclic performances of pure B for half-cell.**

**
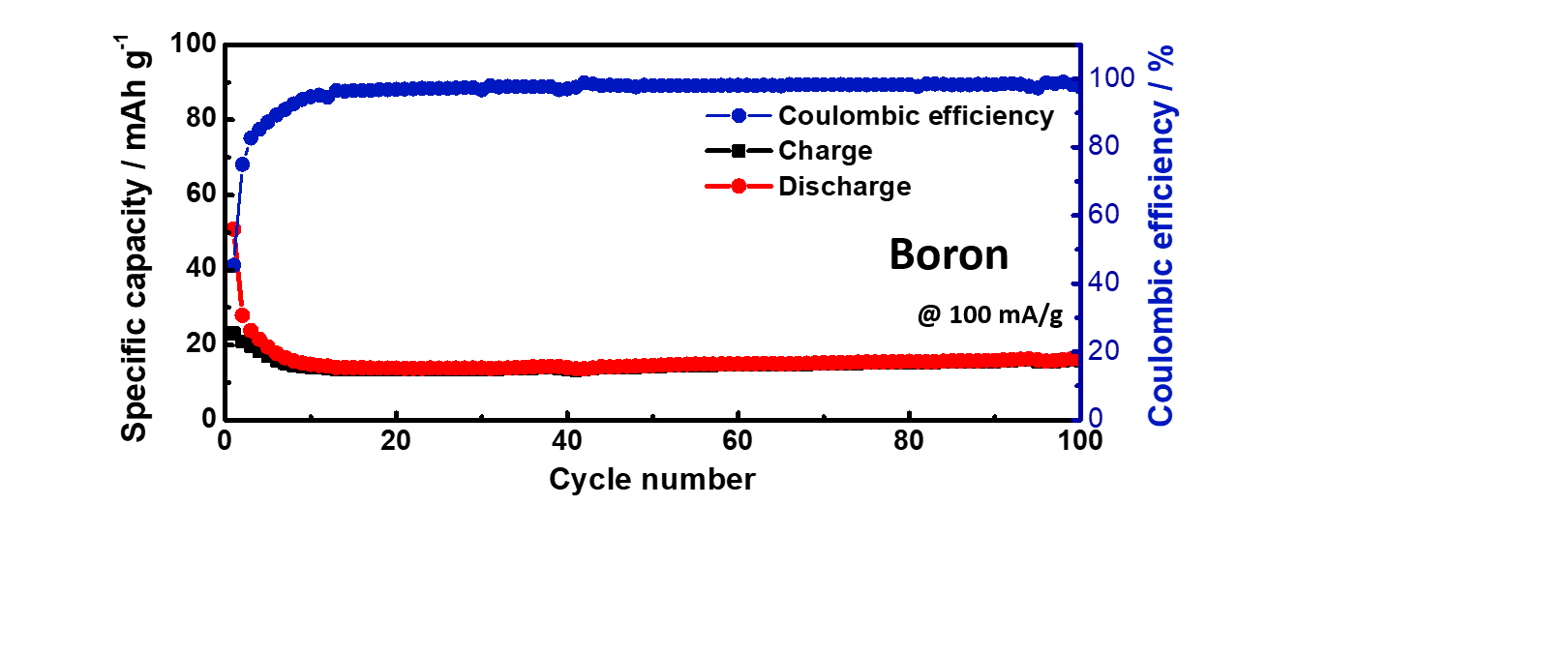
**

**Fig. S2. The charge**–**discharge curves of (a) Ni-B/RGO, (b) Ni-B, and (c) B:** The charge–discharge test was conducted in a range of 0.05–3.0 V_Li_^+^_/Li_.

**
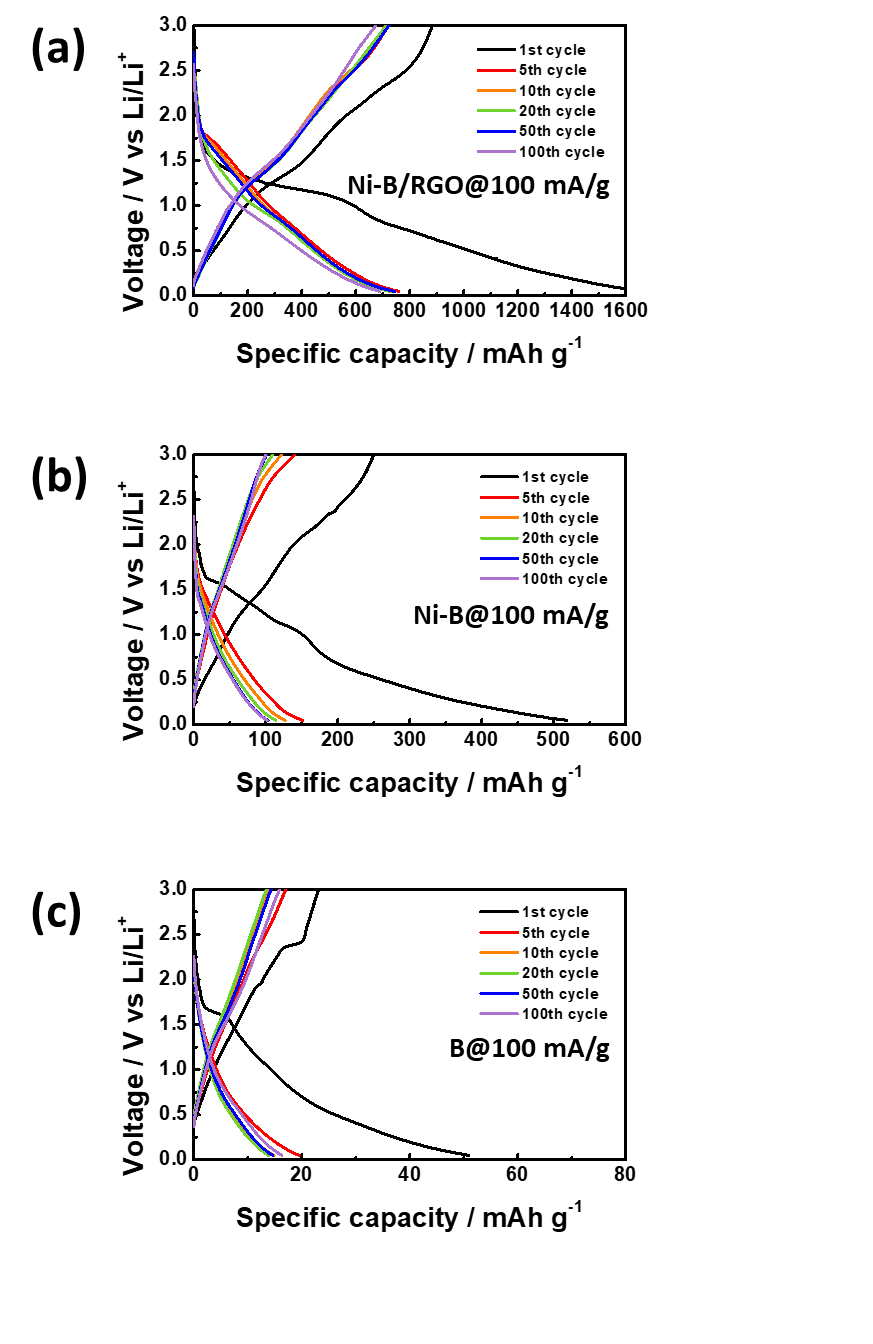
**

**Fig. S3. Cyclic voltammetry (CV) of (a) Ni-B and (b) B half-cells for the initial 3 cycles:** CV was conducted with an operating voltage of 0.05–3.0 V_Li_^+^_/Li_ at 0.1 mV s^-1^.


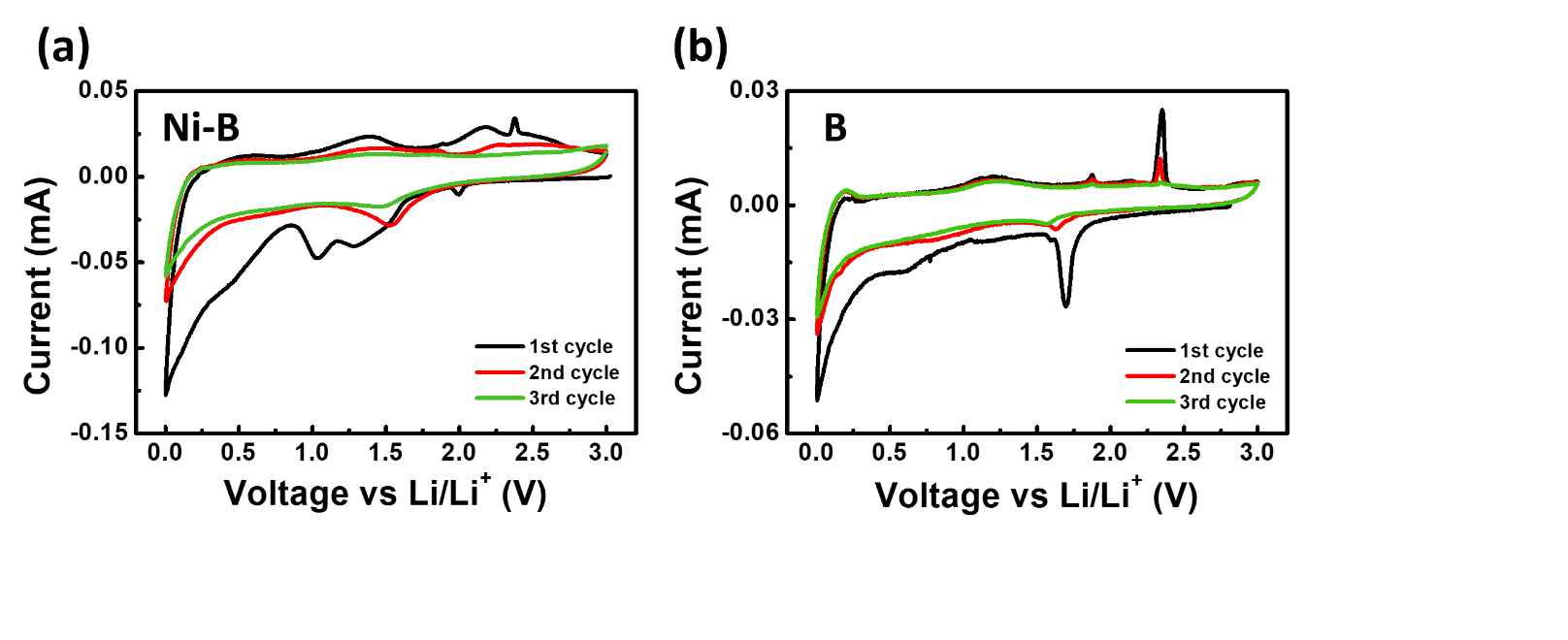


**Fig. S4. Raman spectra of GO and Ni-B/RGO materials**

**
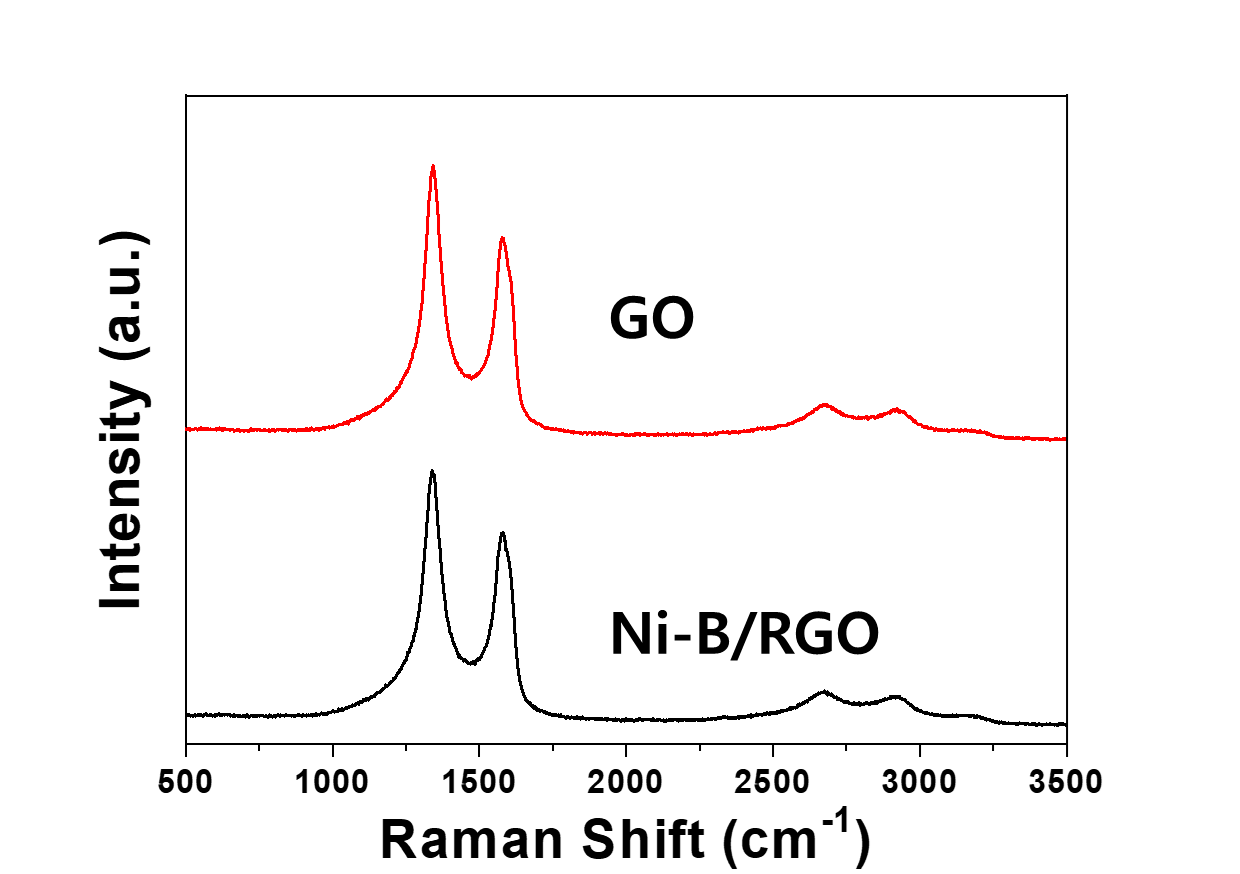
**

1. *K. Eom (keom@gist.ac.kr) and J.-M. Yun (yjm90@kaeri.re.kr) are co-corresponding authors.*

   *G. Im and D. Yun have contributed equally to this work.* [↑](#footnote-ref-1)
